# Temporal dynamics of intradermal cytokine response to tuberculin in *Mycobacterium bovis* BCG-vaccinated cattle using sampling microneedles

**DOI:** 10.1038/s41598-021-86398-6

**Published:** 2021-03-29

**Authors:** Sabine Steinbach, Sasan Jalili-Firoozinezhad, Sreenidhi Srinivasan, Mariane B. Melo, Sonya Middleton, Timm Konold, Michael Coad, Paula T. Hammond, Darrell J. Irvine, Martin Vordermeier, Vivek Kapur

**Affiliations:** 1 Animal and Plant Health Agency, Weybridge, Surrey UK; 2 Koch Institute for Integrative Cancer Research, MIT, Cambridge, MA 02139 USA; 3 Department of Biological Engineering, Massachusetts Institute of Technology, Cambridge, MA 02139 USA; 4 Department of Animal Sciences, Pennsylvania State University, University Park, PA USA; 5 The Huck Institutes of Life Sciences, The Pennsylvania State University, University Park, PA USA; 6 Pathology Department, Animal and Plant Health Agency, Weybridge, Surrey UK; 7 Department of Chemical Engineering, Massachusetts Institute of Technology, Cambridge, MA 02139 USA; 8 Ragon Institute of Massachusetts General Hospital, Massachusetts Institute of Technology and Harvard University, Cambridge, MA 02139 USA; 9 Department of Materials Science and Engineering, Massachusetts Institute of Technology, Cambridge, MA 02139 USA; 10 Howard Hughes Medical Institute, Chevy Chase, MD 20815 USA; 11 Centre for Bovine Tuberculosis, Institute for Biological, Environmental and Rural Sciences, University of Aberystwyth, Aberystwyth, UK

**Keywords:** Immunological techniques, Infectious diseases

## Abstract

Bovine tuberculosis (bTB) is a disease of livestock with severe and worldwide economic, animal welfare and zoonotic consequences. Application of test-and-slaughter-based control polices reliant on tuberculin skin testing has been the mainstay of bTB control in cattle. However, little is known about the temporal development of the bovine tuberculin skin test response at the dermal sites of antigen injection. To fill this knowledge gap, we applied minimally-invasive sampling microneedles (SMNs) for intradermal sampling of interstitial fluid at the tuberculin skin test sites in *Mycobacterium bovis* BCG-vaccinated calves and determined the temporal dynamics of a panel of 15 cytokines and chemokines in situ and in the peripheral blood. The results reveal an orchestrated and coordinated cytokine and local chemokine response, identified IL-1RA as a potential soluble biomarker of a positive tuberculin skin response, and confirmed the utility of IFN-γ and IP-10 for bTB detection in blood-based assays. Together, the results highlight the utility of SMNs to identify novel biomarkers and provide mechanistic insights on the intradermal cytokine and chemokine responses associated with the tuberculin skin test in BCG-sensitized cattle.

## Introduction

Bovine Tuberculosis (bTB) is a disease of economic importance in livestock species such as cattle, sheep and goats and world-wide in distribution. Bovine tuberculosis can be caused by any member of the *Mycobacterium tuberculosis* complex including *M. tuberculosis* (the main causative agent of TB in humans), although in many countries such as the United Kingdom, bTB is caused almost exclusively from infection with *M. bovis*^1^. The disease is an important cause of economic losses to livestock production through lost productivity and the cost of control programmes, and are estimated to be around US$ 3 billion per year^2^. Importantly, bTB has considerable zoonotic relevance, as recently highlighted by the World Health Organization (WHO), World Organisation for Animal Health (OIE) and the Union against Tuberculosis and Lung Disease^3^.

In many high-income countries, bTB is well-controlled or has been largely eliminated by implementation of rigorous test-and-slaugher-based approaches based on active surveillance using tuberculin-based skin testing and slaughter of test-positive animals. Various forms of the tuberculin skin test are employed to test cattle. For example, bTB surveilance in the UK is performed with the comparative cervical tuberculin test (CCT, also referred to as single intradermal compartive cervical tuberculin test, SICCT) based on the comparative responses to intradermal innoculation of a purified protein derative of tuberculin (PPD) prepared from *M. avium* (avian PPD, PPD-A) and PPD prepared from *M. bovis* (bovine PPD, PPD-B). The inclusion of PPD-A assesses background sensitisation of animals with, *e.g.*, environmental mycobacteria to increase test specificity. Other tests include the single cervical tuberculin test (SCT, also referred to as single intradermal test, SIT) that relies on the injection of PPD-B only and is often used as the prescribed test for international trade. Recent developments in tuberculin skin testing for cattle include the use of defined skin test antigen preparations to increase test sensitivity, for example, DIVA tests compatible with BCG vaccination^1, 4–6^.

Whilst tuberculin skin testing of cattle has led to the control and eradication of bTB in many countries, few studies^7^ have aimed to study the temporal development of tuberculin-induced injection skin responses and none has evaluated the kinetics of local cytokine and chemokine responses directly at the local sites of PPD injection that drive the hypersensitivity reaction development. Such information would not only allow mechanistic insights into the development of tuberculin skin test reactivity in bovines, but could also lead to improved diagnostic cytokine read-out systems, particularly when local responses can be compared to systemic responses. With these objectives in mind, we sought a minimally-invasive method to collect interstitial fluid directly from skin test sites, thereby obviating the need for serial biopsies from a large number of animals and minimizing animal welfare concerns.

Minimally invasive approaches for skin interstitial fluid^8^ (ISF) sampling have emerged that are based on compact patches of microneedles^9–14^. Microneedle patches are fabricated in various geometries (solid, porous or hollow) and materials (glass, metal, silicon or polymer) using microfabrication techniques^15^. Here we developed microneedle skin patches capable of effectively penetrating bovine skin and sampling ISF through an absorptive hydrogel coating. These patches enable ISF sampling in a minimally invasive manner. Applying this technology, we obtained serial ISF samples from tuberculin skin test sites on *M. bovis* BCG-vaccinated calves and established chemokine/cytokine kinetic profiles using a recently developed bovine cytokine/chemokine Luminex multiplex array^16^. Taken together, the results reveal a coordinated pro- and anti-inflammatory cytokine and chemokine response during the development of the bovine tuberculin skin test reaction and identify potential biomarkers of a positive tuberculin skin test response.

## Results

### Sampling microneedles can be used for the recovery of skin-resident biomarkers in cattle

**Figure 1:**
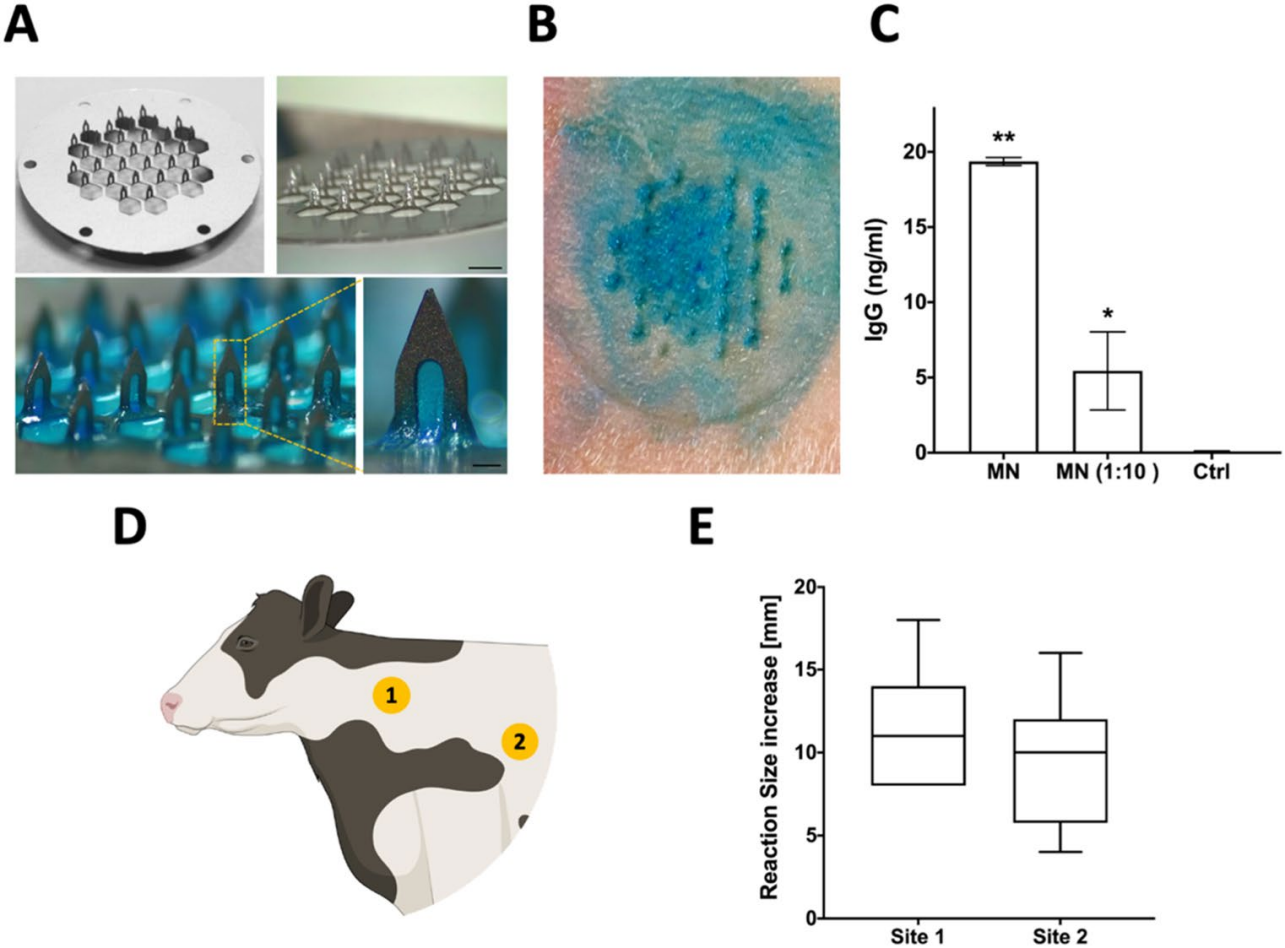
Sampling microneedles enable biomarker analysis from skin explants. (**A**) Medical grade stainless steel microneedle patch (top left) was attached to a PTFE/Silicone support (top right; Scale bar 3 mm) and coated with alginate-based hydrogel (bottom; inset shows the accumulation of alginate in the needle grooves; Scale bar 200 µm). Alginate was mixed with Evans blue dye for better visualization. (**B**) Trypan blue staining post-application confirmed that sampling microneedles efficiently penetrated skin explants. (**C**) IgG concentration as quantified from ISF collected by sampling microneedles. Data shown are means ± SD from one representative of two independent experiments. MN: Sampling microneedle; MN (1:10): Collected ISF was diluted 1:10 in PBS; Ctrl: Sampling microneedle before skin insertion. Statistical analysis: Kruskal–Wallis with Tukey’s multiple comparison test relative to the Ctrl value: *, *P* < 0.05; **, *P* < 0.005. (**D**) Schematic illustration of Site 1 (standard side to inject PPD during single cervical tuberculin test on young calves) and Site 2 (site of SMN application) on the neck of vaccinated calves. (**E**) PPD-B induced skin test reactivity on site 1 and site 2 of five BCG vaccinated calves. Data expressed as increase in reaction size (mm) 82 h following PPD-B injection.

Steel microneedles with a length of 1400 µm were chosen to enable penetration through the cow skin epidermis to the underlying dermis^17^, and mounted on a Teflon disk for handling (Fig. 1A, upper panels). The microneedle array was coated with a biocompatible alginate-based hydrogel to enable sampling of the interstitial fluid at the site of insertion: an alginate/sucrose solution was dried on the microneedle tines, crosslinked by addition of calcium solution, and dried as previously described^18^. High molecular weight (150–250 kDa) alginate was used because of its optimal mechanical and handling properties^9^. Sucrose was included to increase the mechanical resilience of the alginate layer during insertion into the stratum corneum and to enhance the swelling properties of the sampling microneedles (SMNs). Inclusion of Evans blue dye to visualize the coating showed that the resulting patches had a conformal layer of alginate spanning the gaps in each microneedle tine (Fig. 1A). Trypan blue staining confirmed that alginate/sucrose-coated microneedles efficiently penetrated cow skin explants following manual application of the patches (Fig. 1B), The data obtained from skin explant experiments showed that bovine IgG was easily detected (~ 20 ng ml^−1^) in microneedle-recovered ISF from cow skin explants (Fig. 1C), suggesting that SMNs may enable the detection of intradermal biomarkers in cattle.

### Development of in vivo SMN sampling protocols in bovine calves

Since the studies with skin explants demonstrated the feasibility of SMNs to sample bovine skin, we next assessed the potential application of SMNs to naïve or BCG-immunized calves. Our preliminary studies revealed that SMNs could be readily applied with minimal pressure to closely shaved application sites at the sides of the neck of live animals. This pilot in vivo experiment also did not reveal any adverse reactions that might be of animal welfare concern. Indeed, the animals did not demonstrate any visible reaction to SMN application (data not shown). It was noteworthy that the frequent movement of the animals’ heads and neck precluded extended application of the SMNs to the typical tuberculin skin test sites in the cervical region of cattle (Site 1 on Fig. 1D) without the need for additional restraint. In contrast, application of the SMN closer to the shoulder (site 2, Fig. 1D) enabled in situ maintenance of the SMNs for up to 10 min with gentle digital pressure and precluded the need for additional restraint other than a crush. Using this approach, pilot investigations were able to easily detect IgG in ISF samples eluted from SMN inserted into naïve calves and held in situ for 10 min (data not shown). Since the site of inoculation is known to influence the amplitude of the tuberculin skin response^19^, we evaluated whether the shift of injection and sampling sites from site 1, the standard injection sites for tuberculin skin testing, to site 2 still maintained strong skin test reactivity in BCG vaccinated calves. The results showed that the increase in skin thickness at site 2 was numerically but not statistically lower compared to PPD-B induced reactivity at site 1 (Fig. 1E). Thus, for subsequent studies in BCG-immunized cattle calves, tuberculin tests were performed at site 2 and SMNs applied to the same site for 10 min to temporally sample ISF from the area of the developing skin test reaction.

### Temporal development of cytokine/chemokine profiles in ISF samples from tuberculin skin test sites in BCG-immunized cattle

**Figure 2:**
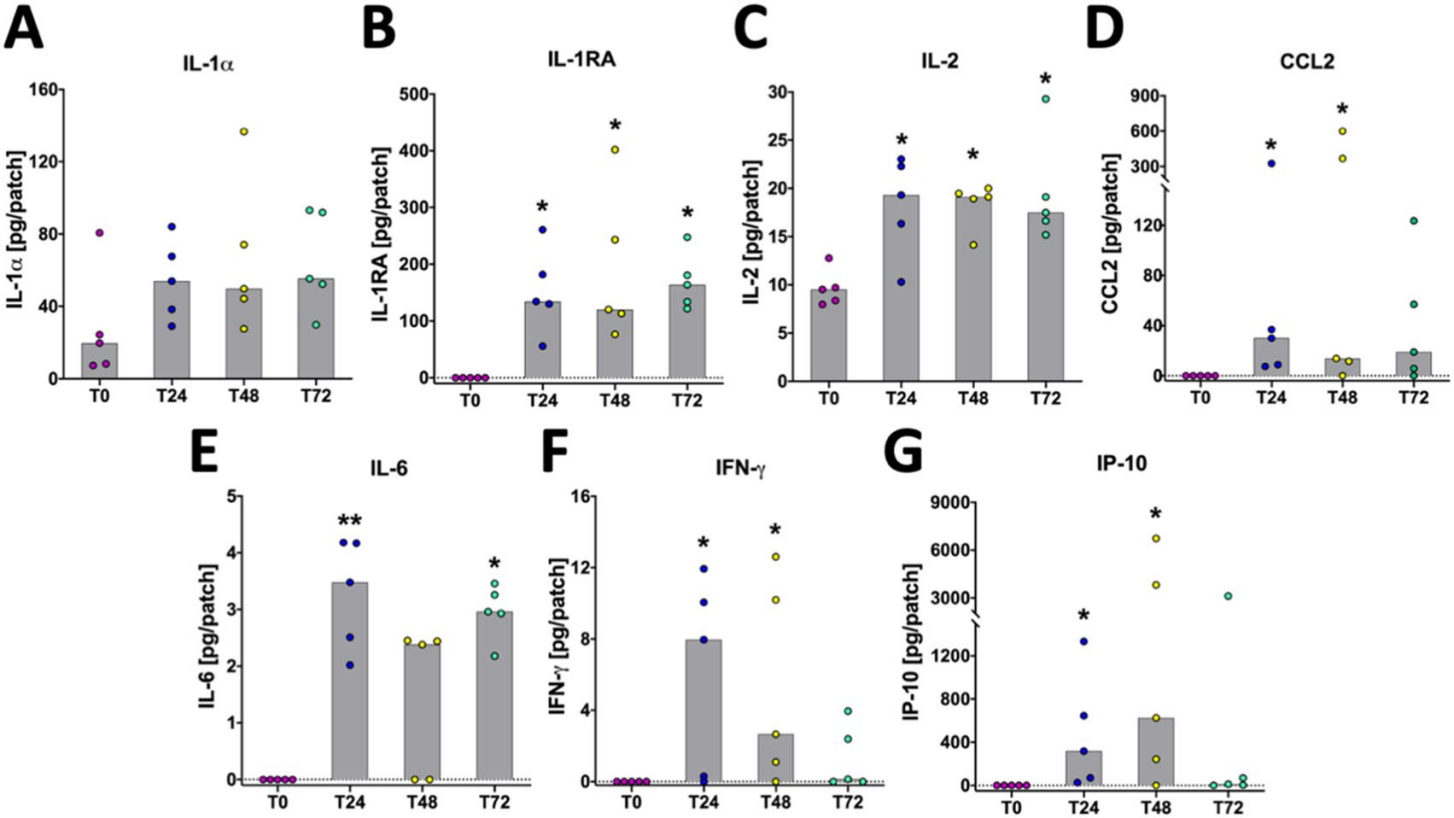
Kinetics of cytokine/chemokine profile in ISF collected from skin test sites of BCG vaccinated calves. ISF was collected prior to (T0) and 24, 48, and 72 h after injection of PPD-B (T24, T48, T72). Cytokine and chemokine content in ISF was determined using a bovine multiplex array via Luminex. Each dot represents the results of individual animal, with bars indicating median responses. Statistical analysis: Kruskal–Wallis with Dunn’s multiple comparison test relative to the T0 values: *, *P* < 0.05; **, *P* < 0.005.

To determine the dynamics of the cytokine/chemokine response in ISF samples collected via SMN, a Luminex-based bovine cytokine/chemokine array detecting 15 bovine cytokines and chemokines was applied. From this assay, 7 cytokines and chemokines (IL-1α, IL-6, IL-1RA, IFN-γ, IL-2, IP-10, CCL2) were detected in the ISF of BCG-immunized calves (Fig. 2A–G). With the exception of IL-1α each of these cytokines and chemokines exhibited a statistically significant (*P* < 0.05) increase in levels in the ISF post-tuberculin injection. The results suggest a coordinated response consisting of induction of early (T24) and sustained (T48, T72) pro-inflammatory cytokine responses; IL-6 showed rapid and sustained expression compared to pre-injection levels and we observed a strong, yet statistically not significant trend (P_T24_ = 0.557, P_T48_ = 0.257 and P_T72_ = 0.296 relative to T0) of increased IL-1α (Fig. 2A,E), each of which is potentially modulated by the early and sustained (T24 through T72) induction of the anti-inflammatory cytokine IL-1RA (Fig. 2B). We further observed a rapid (T24) and significant induction post-tuberculin injection of the chemokines CCL2/MCP1 and IP-10/CXCL10 (*P* < 0.05; Fig. 2D,G). Both chemokines were induced early post PPD-B at T24, but IP-10 peaked at T48 and reverted to pre-PPD-B injection levels at T72 (Fig. 2G). Interestingly, a similar pattern was also observed for IFN-γ whose detection peaked at T24 and decreased towards pre-injection levels by T72 (Fig. 2F). Finally, the results show significant amounts of IL-2 detected at all three post-injection time points (*P* < 0.05; Fig. 2C). None of the other cytokines and chemokines part of the multiplex array (IL-1β, IL-4, IL-8, IL-10, IL-17A, CCL3 (MIP-1α), CCL4 (MIP-1ß), TNF-α) were detectable above the assay limit of quantification (not shown).

**Figure 3:**
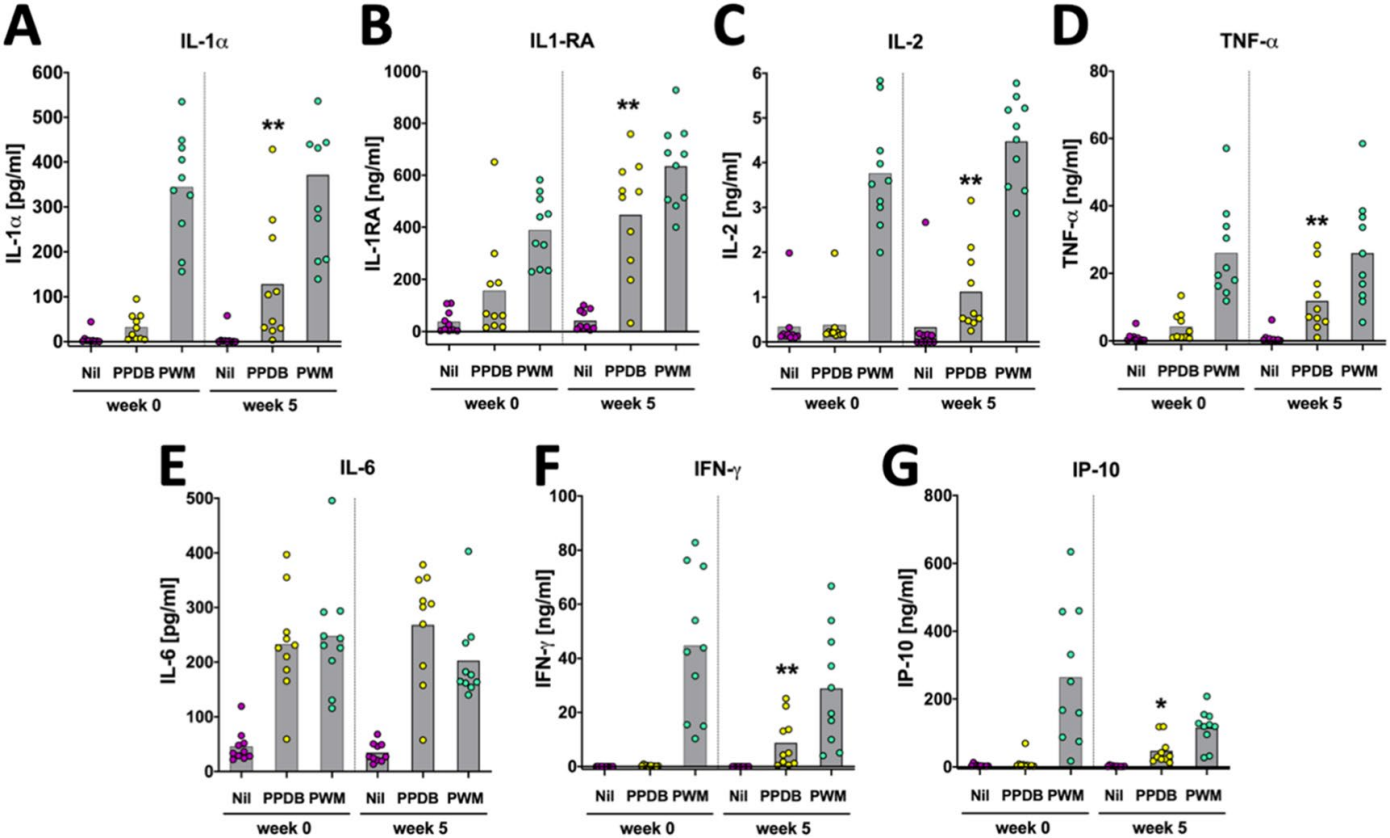
Cytokine and chemokine profiles in whole blood cultures of BCG vaccinated calves before and 5 weeks post-vaccination. Whole blood samples were collected at weeks 0 and 5 post-BCG vaccination and stimulated with PPD-B, PWM or left unstimulated. Cytokine and chemokine profiles were determined using a Luminex-based multiplex array. Each dot represents responses of individual animals with bars indicating mean responses. Statistical analysis: Paired two-tailed t-test: *, *P* < 0.05; **, *P* < 0.005; compared to week 0.

To establish whether these local cytokine/chemokine responses were also reflected by a systemic blood response, whole blood cultures (harvested at weeks 0 (pre-vaccination) and 5 post-BCG vaccination (prior to PPD-B injection)) were stimulated in vitro with PPD-B, with Pokeweed Mitogen (PWM) as a positive control. The cytokine/chemokine responses were determined in plasma supernatants using the bovine 15 plex array described above. Statistically significant increases post-vaccination were detected for IL-1α, IL-1RA, IFN-γ IL-2, IP-10 and TNF-α (*P* < 0.05, Fig. 3A-D, F, G). IL-6 production was also increased post-vaccination although its plasma level increase did not reach statistical significance (Fig. 3E). In contrast, no, or only sporadic and insignificant antigen-specific IL-1β, IL-4, IL-10, or IL-17A responses were detectable (data not shown). Responses to CCL2, CCL3, CCL4 and IL-8 could not be measured in plasma due to high levels of interference (data not shown). To conclude, the cytokine/chemokine profiles at the skin sites of PPD-B injection are largely reflected in the PPD-B stimulated blood responses, with the exception of TNF-α which was not detected in ISF obtained from SMNs.

## Discussion

Interstitial fluid contains a variety of disease- and immune status-associated molecular biomarkers^20, 21^. A microneedle-based platform can be utilized to sample skin ISF in a pain-free manner, from several hundred microns under the skin surface, where only a few capillary beds and pain receptors reside in the connective tissue dermis. We here explored this promising sampling approach in order to begin to characterize functional aspects of cytokine and chemokine infiltrates, and also accurately monitor temporal immune reactions at the dermal sites of tuberculin PPD injection to unravel key tissue-resident immune biomarkers to help improve bTB diagnosis^13^. Data thereby generated not only provide mechanistic insights into the way bovine tuberculin skin test reactions develop but these data could also play an important role in the identification of novel diagnostic biomarkers for accurate diagnosis.

Immune monitoring approaches in humans and animal models primarily rely on peripheral blood analysis, which can fail to report on the status of major immune cell populations, chemokines and cytokines localized in peripheral tissues including barrier tissues such as the skin^22, 23^. Skin ISF has been mostly overlooked despite containing a variety of disease- and immune status-associated biomarkers^20, 21^. While in this study we concentrated on collecting ISF, in small animal models, collection and functional characterization of skin resident T cell population has been demonstrated^9^. This, however, required the SMN to be in contact with the dermis for extended periods (hours) in contrast with the 10-min contact time used in the current study on calves. Such extended SMN-skin contact periods in large animals can be more challenging, especially because restraining the animals for a prolonged duration is a potential animal welfare concern, and hence may require the development of alternative approaches to ensure the SMNs remain in situ in unrestrained animals through future studies focused on an improved SMN design that enables cell sampling. Apart from sampling skin sites, the microneedles can also be modified to deliver diagnostic antigens. Pilot data suggest that peptide antigens bound to microneedle patch design will be quickly eluted after the needles had been applied to cow skin (unpublished data).

**Figure 4:**
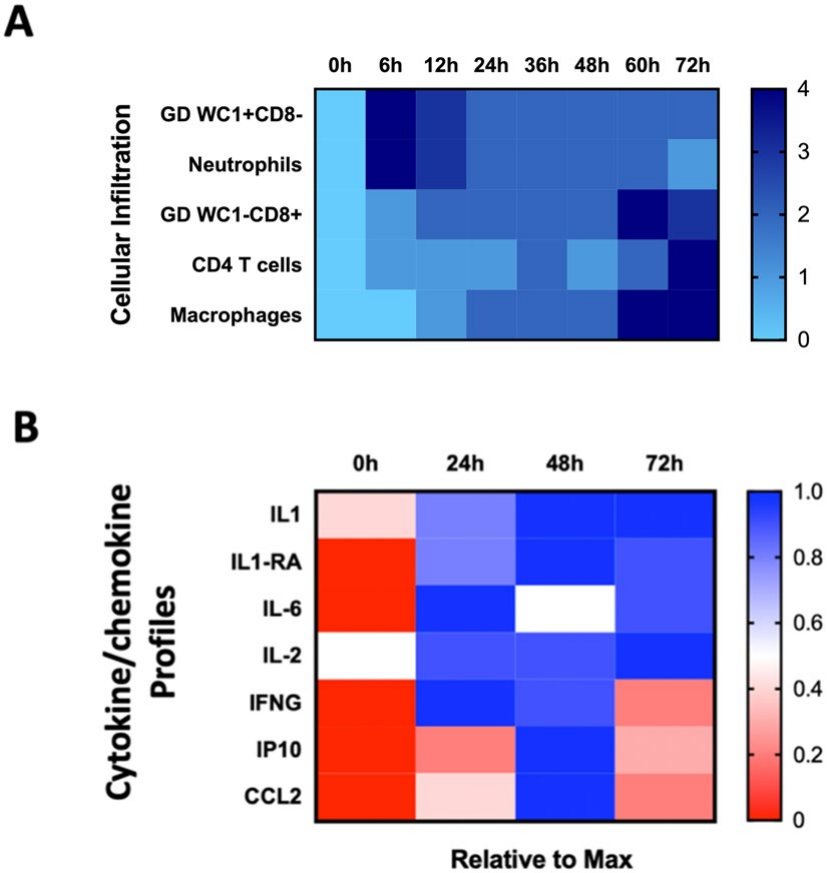
Schematic representation of the kinetics of cellular accumulation (**A**) and cytokine/chemokine profiles (**B**) in bovine tuberculin skin test reaction. (**A**) Cellular compost profiles according to Doherty et al^7^. Cell subset accumulation was scored from 0 to 4 (maximum response) and is expressed as heat map. (**B**) Biomarker profiles established in the present study are expressed as heat-map based on the maximum expression (100%) of each chemokine and cytokine represented.

The current study is the first to describe the kinetics of cytokine/chemokine responses in ISF sampled directly from bovine tuberculin skin test sites. To bring these cytokine and chemokine profiles into context with other time-course data generated during the development of tuberculin skin test reactions is made challenging due to a relative paucity of prior literature. However, a study by Doherty et al. in the late 1990s, applying immunohistochemistry approaches to define the cellular subsets in skin biopsies taken during the 72 h post-PPD injection, provides some context and their findings are summarised in Fig. 4A^7^. They documented a complex and coordinated kinetic of cell populations following tuberculin injection: early (within first 5–12 h) influx of γδ T cells expressing WC1 but not CD8, as well as early accumulation of neutrophils followed later (between 36 and 72 h) by accumulation of CD4 + T cells and macrophages, both reaching their maximum levels at the 72 h sampling point. Interestingly, they also showed evidence of a role for a second γδ T cell population (WC1-CD8+), increasing in the reaction sites from 24 h onwards and reaching their maximum level at the end of the observation period at 72 h post-PPD-B injection (Fig. 4A). Similarly, in the current study cytokine/chemokine profiles post-tuberculin injection is also indicative of a coordinated and choreographed response kinetic (Fig. 4B) that can be reconciled with the cellular composition data in Fig. 4A. It was observed that IP-10 and CCL2 levels rise at T24, peak at T48 and then decline at T72. As both have roles in supporting the recruitment of macrophages/monocytes from the vasculature (CCL2) and activate T cells and macrophages (IP-10), their accumulation in skin test sites at T24 and later is likely to be instrumental in the recruitment of T cells and macrophages as described by Doherty et al. The sustained expression of pro-inflammatory cytokines like IL-6 whose functions include T cell and macrophage activation, is also likely to enhance the T cell and macrophage activity involved in this delayed-type hypersensitivity (DTH) tuberculin-induced reaction. Interestingly, IFN-γ levels peaked already at T24 and then decreased to almost pre-injection levels at T72. This could suggest that this early peak of this important cytokine might not be solely caused by CD4^+^ T cells as they only start to accumulate in the reaction sites by 48 h and later post-tuberculin injection, although the level of cytokine production per individual cell will also depend on its activation status. Highly activated tissue-resident CD4^+^ T cells could also explain this early peak of IFN-γ responses. Alternatively, it is also conceivable that this rapid increase in IFN- γ levels at T24 is due to its production by the first wave of γδ T cells accumulating rapidly after tuberculin injection (Fig. 4A). Another possible source of IFN-γ production could also be Natural Killer (NK) cells, whose presence in skin test sites was not assessed by Doherty et al. The increased and sustained prediction of IL-2 at T24 to T72 is also indicative of on-going T cell activation and proliferation.

We also found evidence for a mechanism to limit/control the inflammatory responses in the skin test sites as we found increased levels of the anti-inflammatory cytokine IL-1RA from T24 to T72. Doherty et al., speculated on a regulatory role in limiting the inflammation of the second γδT cell population (WC1-CD8 +) that started to accumulate in the skin test sites from 24 h post-PPD-B injection onwards (Fig. 4A). Although they could not demonstrate this directly, it is a plausible explanation as bovine γδT cells can carry T regulatory cell functions^24, 25^. Interestingly, antigen-specific IL1RA gene expression had been recently documented by Palmer et al. in whole blood cells from calves infected with *M. bovis*^26^, and the present study is the first to report PPD-specific IL-1RA production at the protein level locally and systemically. This analyte could constitute a useful novel diagnostic biomarker suitable for bTB blood tests. We did not detect increased levels of TNF-α, which is a cytokine commonly associated with DTH reactions in bovine tuberculin reaction sites. We compared the local cytokine/chemokine profiles with responses induced in vitro before and 5 weeks after vaccination and demonstrated that the local response profiles were almost completely reflective of systemic blood cell responses with the exception of TNF-α which we could only demonstrate in PPD-B stimulated whole blood cell cultures.

Studies on transcriptional profiling of human tuberculin (Mantoux) skin reactions has shown dominant expression of genes associated with T cell activation and recruitment. In addition, systemic responses in humans were also reflected in those observed at skin test reaction sites. Innate immune gene expression characterised by chemokines were detected including CCL2 and IP-10 that were also featuring prominently in our study^27–29^. However, unlike the present study, they did not report anti-inflammatory IL-1RA responses potentially regulating and limiting the tissue damage and inflammation in the tuberculin skin test reaction sites in cattle.

Our studies showed several cytokines and chemokines that remained undetectable (or were only infrequently detected and at low concentrations so as to preclude statistical analyses) in blood and interstitial fluid, in particular IL-1β, IL-4, IL-10 and IL-17A. In addition, CCL3 and CCL4 had high plasma background levels even prior to vaccination and in the absence of stimulus and hence precluded analysis of BCG vaccination or antigen-specific responses. The failure to detect an IL-4 response is consistent with previous findings in naturally infected cattle^30^. Indeed, in a previous study in calves, an increased post-*M. bovis* infection expression of the splice variant IL-4δ833, an IL-4 antagonist, which presumably contributes to a stronger TH1 responses was noted^31^. IL-10 responses have been described post-infection of cattle. However, the amount of PPD-specific IL-10 was associated with reduced skin test activity and disease progression^32^. Since BCG vaccination likely results in non-pathogenic outcomes in cattle, perhaps the failure to detect measurable levels in ISF samples of this cytokine in the current studies is consistent with IL-10 being a marker of disease progression but not immunity or protection.

Interleukin-1 induction is a signature response of trained innate immunity post-BCG vaccination^33^ in humans and small animal models and is consistent with our detection of IL-1α both in blood and ISF in the present study. In contrast, the absence of IL-1β both in ISF and the whole blood cultures stimulated with PPD was unexpected as this cytokine has previously been considered as a potential biomarker for bovine tuberculosis diagnosis^30^ as well as shown to be elevated with positive tuberculin skin test in naturally infected cattle^32^. While IL-1β responses following BCG vaccination in cattle are unknown and require further investigation, the short post-vaccination period between vaccination and sampling the animals may also have resulted in animals not having fully developed all of the features of trained immunity.

Expression of IL-17A has been described in several studies as correlate of protection post-vaccination, with strong responses observed in animals vaccinated with BCG/adenoviral heterologous prime-boost approaches^34^ or genetically improved BCG strains^35^. Furthermore, increased expression of IL-17A has also been described as biomarker of *M. bovis* infected cattle^36, 37^. However, the detection of IL-17A protein has been shown to be less sensitive than detection of its transcript, particularly post-BCG vaccination^38^. In contrast, antigen-specific IL-17A protein can be more readily detected post-infection^38, 39^. Hence, the infrequent and only low level detection of antigen-specific IL-17A in the current study in whole blood cultures from BCG vaccinated animals is not altogether unexpected, and additional studies in *M. bovis* infected cattle are warranted to better define the role and potential of this and other cytokines as a potential biomarker using the SMN approach.

In conclusion, the results of our investigations suggest that the minimally invasive microneedle-based sampling approach enables characterization of chemokine and cytokine profiles in ISF from the dermal reaction sites in BCG-vaccinated cattle. Despite the limitations of being a proof-of-concept study with small sample sizes and findings that need to be confirmed in TB infected cattle, the results reveal an orchestrated and coordinated cytokine and local chemokine response and provide mechanistic insights on potential local regulation of skin test inflammation in response to tuberculin. The identification of IL-1RA as a potential diagnostic blood test biomarker and confirmation of the utility of biomarkers such as IFN-γ and IP-10 for bTB detection in blood-based assays suggest that the SMN approach may be of utility in future investigations for defining key immune signatures associated with the development of the Type IV response to tuberculin in cattle and for discovery and validation of novel biomarkers for improved bTB diagnosis.

## Methods

### Fabrication of sampling microneedles

The solid metal microneedle patch (AdminPatch 1500, AdminMed, NanoBiosciences LLC, California, USA) has 31 needle tines, each 1400 µm in length, distributed as a diamond shape on a 1 cm^2^ circular area. The entire patch is 20 mm in diameter and is fabricated from medical grade stainless steel. A custom-made PTFE/Silicone (Septa 27236, Sigma) support was attached to the back of each microneedle patch using a super glue for ease of handling and to promote uniform insertion of needles into skin samples. To generate SMN patches, needles were functionalized with an alginate-based hydrogel coating as described previously^10^. In brief, after oxygen plasma treatment (10 min, 18 W oxygen plasma produced by a PDC-32G plasma cleaner), 200 µl of a 0.01 wt% solution of poly(L-lysine) (150–300 kDa, Sigma, P4832) was pipetted and dried onto each microneedle to form an adsorbed layer that will subsequently facilitate electrostatic adhesion of the alginate hydrogel. A volume of 200 µl of alginate solution (1.16 mg of alginate (MW: 150–250 kDa, Viscosity: 100–300 mPa.s, G/M Ratio: ≥ 1, Endotoxins: ≤ 100 EU/g; PRONOVA SLG100, FMC BioPolymer) and 4.66 mg of sucrose (Sigma) in 200 µl of water) was pipetted onto each microneedle array and air dried at 25 °C for at least 4 h. Finally, 200 µl of crosslinking solution (20 mM CaCl_2_) was pipetted onto each patch and dried at 25 °C for at least 12 h. To visualize the sampling layer, alginate was mixed with 10 µl of Evans blue dye (10 mg/ml, Sigma) before coating the microneedle array.

### SMN application to bovine skin tissue explants

Skin tissue samples from the neck region (same as tuberculin injection site) of animals were obtained from an USDA-inspected slaughterhouse in Central Pennsylvania. The tissue was briefly washed with 70% ethanol on site and then transported in PBS (on ice) to the laboratory. Upon arriving in the laboratory, tissue was preserved in DMEM (with Penicillin–Streptomycin) at 4 °C until use. After shaving the skin explants, SMNs were pressed onto explanted skin by applying gentle digital pressure, and adherence maintained using 3 M Nexcare waterproof tape. Treated skin was stained with trypan blue for needle penetration. After 30 min at 37 °C, SMNs were gently removed from the skin explant and immersed in 200 µl of PBS containing 100 mM EDTA and incubated at 37 °C on a shaker at 150 rpm for 30 min. The supernatant was collected and quantified via enzyme-linked immunosorbent assay (ELISA, R&D Systems, DY5930) for immunoglobulin G (IgG).

### BCG immunization of bovine calves

Experiments were conducted under a UK Home Office project license granted under the Animal (Scientific Procedures) Act and following approval of the experiments by the local Animal Welfare and Ethical Review Board. All animal procedures at Animal and Plant Health Agency (APHA) were approved by the APHA Animal Welfare and Ethical Review Board (AWERB), in accordance with relevant guidelines and regulations, and all authors complied with the ARRIVE guidelines. Calves (3 months old, males, Holstein-Frisian or crosses thereof) were sourced from Great Britain herds in the Low Risk Area that were Officially TB Free. They were vaccinated via the subcutaneous route with 1–4 × 10^6^ CFU BCG Danish SSI strain 1331 (AJ Vaccine, Copenhagen, Denmark). Blood samples were drawn prior to vaccination and 6 weeks after vaccination. Skin testing and collection of ISF by SMNs were performed 6 weeks post-vaccination.

### Single cervical tuberculin skin test (SCT) and sampling procedure

PPD-B (3000 IU/ml, Thermo Fisher, Schlieren, Switzerland) was injected intradermally in the standard SCT site (site 1, Fig. 1D) on the neck of vaccinated calves and increases in skin reaction measured 72 h later. At the time of SCT injection, the same amounts and volumes of PPD-B were injected into site 2 (Fig. 1D) on both sites of the neck. These sites were sampled by applying sampling microneedles for 10 min. Prior to PPD-B injection, sites to be sampled were shaved closely. The animals were randomly placed into two groups of 5 calves. One group was sampled by applying the SMNs to site 2 on one side of the neck without PPD-B injection (T0), and to site 2, which received PPD-B, on the other side of the neck, 48 h following PPD-B injection (T48). The second group of calves was injected with PPD-B into site 2 on both sides of their necks, and sampled in the same way 24 and 72 h post tuberculin injection (T24, T72), ISF was eluted from the SMNs as described above. This injection protocol was set up to ensure that each animal was only sampled once on each site with microneedles. The pilot study demonstrated that the SMNs would not stay in the skin unless some sort of pressure was applied throughout the sampling time.

### Interferon-gamma release assay (IGRA)

Heparinised blood was collected and aliquots of 0.25 ml were plated in 96-well plates. Antigen (0.025 ml/well) was added, namely, bovine PPD (PPD-B, final assay concentration 300 IU/ml), avian PPD (PPD-A, final assay concentration 250 IU/ml), Pokeweed Mitogen as positive control (final assay concertation 10 µg/ml) and a no antigen (medium only) control for 20 h at 37 °C in the presence of 5% CO_2_, following which cell supernatants were removed and stored at − 80 °C until required. Quantification of IFN-γ in whole blood culture supernatant was determined using the commercially available BOVIGAM enzyme-linked immunosorbent assay (ELISA) kit (Thermo Fisher, USA). Results were expressed as the optical density at 450 nm (OD_450_) for cultures stimulated with bovine tuberculin PPD (PPD-B) and the OD_450_ for cultures without antigen.

### Bovine cytokine/chemokine multiplex assay

Cytokines/chemokines in interstitial fluid and plasma samples were quantified using a bovine customized multiplex (MILLIPLEX MAP Custom Bovine Cytokine/Chemokine Magnetic Bead Premixed 15 plex Panel, Merck Millipore, UK) assay kit^16^. Plasma and EDTA-free (after buffer exchange using Zeba Spin Desalting Columns, Thermo Fisher) interstitial fluid samples were thawed, mixed and centrifuged at 1000 g for 10 min. After dilution of plasma (1:2 and 1:10 in assay buffer provided in the kit) and interstitial fluid [1:2 in RPMI 1640/20% FCS (Gibco Life Technologies, UK)] 25 µl of each sample were processed in duplicates (interstitial fluid) or two different dilutions (plasma) according to the manufacturer’s instruction and analysed on Luminex 200 to determine IL-1α, IL-1β, IL-1RA, IL-2, IL-4, IL-6, IL-8, IL-10, IL-17A, IFN-γ, TNF-α, CCL2 (MCP-1), CCL3 (MIP-1α), CCL4 (MIP-1ß) and CXCL10 (IP-10). Belysa immunoassay curve-fitting software was used to calculate concentrations from Median Fluorescent Intensity (MFI) data. Only values ≥ lower limit of quantification (LLoQ; determined by the software) are shown in the graphs. For interstitial fluid samples the total amount of cytokine was calculated by using the final sample volume obtained by elution from sampling microneedles.

### Statistical analysis

Statistical analysis was undertaken using Prism v7 (GraphPad San Diego, CA, USA). IGRA and blood cytokine responses were analysed by paired two-tailed t-tests. Skin test responses between sites 1 analysed by Mann–Whitney U test (two-tailed). Temporal development of cytokine and chemokine responses in ISFs were assessed using the two-tailed Kruskal–Wallis test with Dunn’s post- test application. Significance level (alpha) was set at 5%.

## Supplementary Information

Supplementary Information.

## Data Availability

All data needed to evaluate the conclusions in the paper are present in the paper and/or the Supplementary Materials. Additional data related to this paper may be requested from the authors.
