# Supplementary material for: Temporal dynamics of intradermal cytokine response to tuberculin in Mycobacterium bovis BCG-vaccinated cattle using sampling microneedles
Source: Sci Rep. 2021 Mar 29;11:7074. doi: 10.1038/s41598-021-86398-6 (PMC8007627; doi:10.1038/s41598-021-86398-6)
Supplement: Supplementary file 1 — Supplementary Information. [file 41598_2021_86398_MOESM1_ESM.docx]

**Temporal dynamics of intradermal cytokine response to tuberculin in *Mycobacterium bovis* BCG-vaccinated cattle using sampling microneedles**

Sabine Steinbach^1,†^, Sasan Jalili-Firoozinezhad^2,3,†^, Sreenidhi Srinivasan^5,†^, Mariane B. Melo^2,3^, Sonya Middleton^1^, Timm Konold^6^, Michael Coad^1^, Paula T. Hammond^2,7^, Darrell J Irvine^2,3,8,9,10,*^, Martin Vordermeier^1,11,*^, Vivek Kapur^4,5,*^

^1^Animal and Plant Health Agency, Weybridge, Surrey, UK

^2^Koch Institute for Integrative Cancer Research, MIT, Cambridge, MA 02139, USA

^3^Department of Biological Engineering, Massachusetts Institute of Technology, Cambridge, MA 02139, USA

^4^Department of Animal Sciences, Pennsylvania State University, University Park, PA, USA

^5^The Huck Institutes of Life Sciences, The Pennsylvania State University, University Park, PA, USA

^6^Pathology Department, Animal and Plant Health Agency, Weybridge, Surrey, UK

^7^Department of Chemical Engineering, Massachusetts Institute of Technology, Cambridge, MA, 02139, USA

^8^Ragon Institute of Massachusetts General Hospital, Massachusetts Institute of Technology and Harvard University, Cambridge, MA 02139, USA

^9^Department of Materials Science and Engineering, Massachusetts Institute of Technology, Cambridge, MA 02139, USA

^10^Howard Hughes Medical Institute, Chevy Chase, MD 20815, USA

^11^Centre for Bovine Tuberculosis, Institute for Biological, Environmental and Rural Sciences, University of Aberystwyth, Aberystwyth, UK.

^†^Contributed equally

*Correspondence to: [Martin.Vordermeier@apha.gov.uk](mailto:Martin.Vordermeier@apha.gov.uk), [djirvine@mit.edu](mailto:djirvine@mit.edu), or [vkapur@psu.edu](mailto:vkapur@psu.edu)

**Supplemental data:**

**BCG vaccination experiment.** After demonstrating the utility of SMN to generate ISF from skin test sites, we next performed a BCG vaccination experiment. The objective was to obtain a time-course series of ISF samples directly from tuberculin skin test sites using SMN. Ten calves were vaccinated with a single standard dose of BCG Danish SSI, strain 1331 (1-4 x 10^6^ CFU/dose) via the subcutaneous route. To confirm successful vaccine take, blood was drawn before and 5 weeks post-BCG (*i.e.* prior to skin testing) and stimulated with PPD-A, PPD-B to measure IFN-γ production (IGRA). Both PPD-A and PPD-B-specific IFN-γ responses significantly increased after vaccination with a strong bias towards PPD-B specific IFN-γ responses (**Fig. S1**, P < 0.005), thus confirming vaccine take. Six weeks post-BCG vaccination, PPD-B was injected in the standard tuberculin test sites (site 1) and in sites 2, using available sites 2 on both sides of the neck (*i.e.* 2 injections at site 2 per animal). 72 h later, we observed strong skin responses in all animals for at the standard SCT (Median [range]: 11 mm [6, 19 mm]) further confirming successful vaccination. ISF samples were collected (n=5) prior to and 24, 48 and 72 h post-PPD-B injection from site 2. Five animals were sampled using SMN prior to and 48 h post-PPD-B injection making use of the two site 2 on both sides of the neck (T0, T48). A second group of 5 BCG vaccinated calves were sampled 24 and 72 h post-tuberculin injection (T24, T72).

**
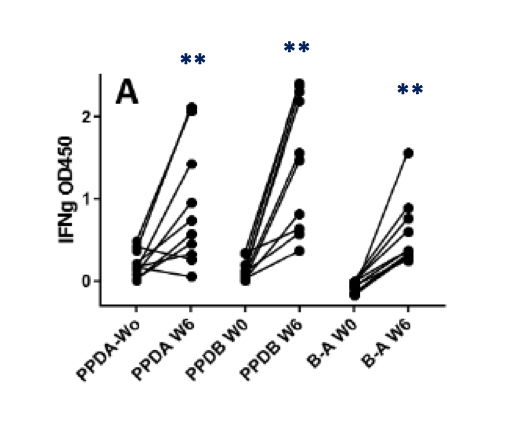
**

**Figure S1. Confirmation of vaccine take in 10 BCG vaccinated calves.** *In vitro* production of IFN-γ after stimulation of whole blood with PPD-A, PPD-B prior to and 5 weeks post-BCG vaccination. Results expressed as ΔOD450 with background values subtracted. The bias towards PPD-B induced responses post-vaccination is demonstrate by the B-A column (OD450 with PPD-A subtracted from OD450 values induced by PPD-B). **, P < 0.005, Paired t-test).
